# Mosquito (Diptera: Culicidae) Habitat Surveillance by Android Mobile Devices in Guangzhou, China

**DOI:** 10.3390/insects7040079

**Published:** 2016-12-17

**Authors:** Tai-Ping Wu, Jun-Hua Tian, Rui-De Xue, Yi-Liang Fang, Ai-Hua Zheng

**Affiliations:** 1 Wuhan Centers for Disease Prevention & Control, Disinfection and Vector Control Section, Wuhan 430015, China; celerwu@hotmail.com (T.-P.W.); tianjunhua1980@163.com (J.-H.T.); 2 Anastasia Mosquito Control District of St. Johns County, 120 EOC Drive, St. Augustine, FL 32092, USA; 3 Fujian International Travel Healthcare Center, Fuzhou 350001, China; Anew627@163.com; 4 The State Key Laboratory of Integrated Management of Pest Insects and Rodents, Institute of Zoology, Chinese Academy of Sciences, Beijing 100101, China; zhengaihua@ioz.ac.cn

**Keywords:** mosquito habitat, *Aedes albopictus*, mosquito surveillance, android mobile device, OruxMaps

## Abstract

In 2014, Guangzhou City, South China, suffered from its worst outbreak of dengue fever in decades. Larval mosquito habitat surveillance was carried out by using android mobile devices in four study sites in May 2015. The habitats with larval mosquitoes were recorded as photo waypoints in OruxMaps or in videos. The total number of potential mosquito habitats was 342, of which 166 (49%) were found to have mosquito larvae or pupae. Small containers were the most abundant potential habitats, accounting for 26% of the total number. More mosquito larvae and pupae, were found in small containers than in other objects holding water, for example, potted or hydroponic plants (*p* < 0.05). Mosquito larvae were collected from all plastic road barriers, used tires, and underground water. *Aedes albopictus* larvae were found from small and large containers, stumps, among others. The overall route index (RI) was 11.3, which was 14.2 times higher than the grade C criteria of the National Patriotic Health Campaign Committee (NPHCC), China. The higher RIs were found from the bird and flower markets, schools, and underground parking lots. The results indicated that Android mobile devices are a convenient and useful tool for surveillance of mosquito habitats, and the enhancement of source reduction may benefit the prevention and control of dengue vector mosquitoes.

## 1. Introduction

Dengue is predominantly an urban disease that is vectored by container-inhabiting *Aedes* mosquitoes [1,2] Guangzhou, the capital city of Guangdong province in south China, has experienced outbreaks of dengue fever for many years [3], and *Aedes albopictus* Skuse has been confirmed as the dengue vector [4]. The most serious outbreak of dengue fever in Guangzhou was from June to December 2014, with a report of 37,331 cases (Emergency Management Office of the People’s Government of Guangdong Province). During the peak of the dengue epidemic in Guangzhou in 2014, a large-scale ground application of adulticiding was conducted, but the source reduction and larval control was neglected.

Larval mosquito surveillance involves sampling a wide range of aquatic habitats for the presence of immature mosquitoes with a dipper or plastic pipette [2,5]. The spatial distribution and abundance of immature mosquito habitats [6] have been usually recorded and analyzed by papers, pictures, Microsoft Excel program, computer models [2], Vector Control Management System databases, and MapVision [7]. Android mobile devices and mobile cell phones are popular tools [8,9], but these tools have not been widely used for surveillance of mosquito habitats.

The objectives of this study were to: (1) determine the distribution and abundance of immature mosquito habitats at the site of the previous dengue outbreak and (2) determine the potential application of the popular Android mobile devices for surveillance of mosquito habitats.

## 2. Materials and Methods

**Figure 1:**
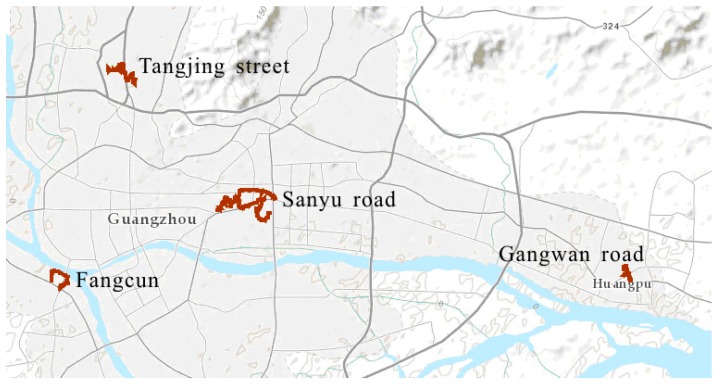
Four study sites (orange lines) in Guangzhou city.

Four sites (Sanyu Road in Yuexiu District, Tangjing Street in Baiyun District, Gangwan Road in Huangpu District, and Fangcun Road in Liwan District, Table 1) in Guangzhou City were selected for the study on the habitat surveillance and inspection route mapping (Figure 1). The last three sites formed a triangle with the first site in the center (Figure 1). The first indigenous dengue case was reported from Tangjing Street in Baiyun District, Guangzhou City, in 2015.

Android mobile devices are very popular in China. Cameras on devices and apps, such as AutoNavi Navigation and Baidu Maps, have been facilitated for immature mosquito surveillance [8]. OruxMaps was a free map-viewing app developed by Jose Vazquez for Android mobile devices that could record routes of inspection [9,10].

A Samsung Galaxy Table 3 (Samsung Electronics, Suwon, South Korea) and OruxMaps, AutoNavi Navigation (AutoNavi Holdings, Beijing, China), and Baidu Maps (Baidu, Beijing, China) were used in this study. Before surveillance, the study sites were checked in Baidu Maps and the inspection routes were planned according to the availability of premise types. Some points on the route were collected in AutoNavi Navigation. Potential habitats were categorized as small containers (less than 37 cm in diameter), large containers (equal to or larger than 37 cm in diameter), stumps (bamboo stumps or the rooted remains of metal tubes), artificial objects (such as leather shoes, toys, etc.), cisterns, puddles, storm drains, shallow water (2–8 cm depth), depressions of tree roots, plastic road barriers, potted or hydroponic plants, fallen leaves, ditches, used tires, and subterranean pits and chambers [11]. Searches for stagnant water were conducted mainly outdoors and tracked in the log of OruxMaps with GPS. Before inspection of a new premises, a picture of the name or feature of the premises was taken. The habitats with immature mosquitoes were recorded as photo waypoints or recorded in videos. Pictures of the habitats that were negative for mosquito larvae and pupae were taken by the Android mobile device for further identification of the habitats. Auxiliary information of mosquito habitats was recorded quickly, and the immature mosquitoes were sampled with a dipper or plastic pipette [2,5] and sent to Fujian International Travel Healthcare Center in Fuzhou for species identification by morphological characters.

**Figure 2:**
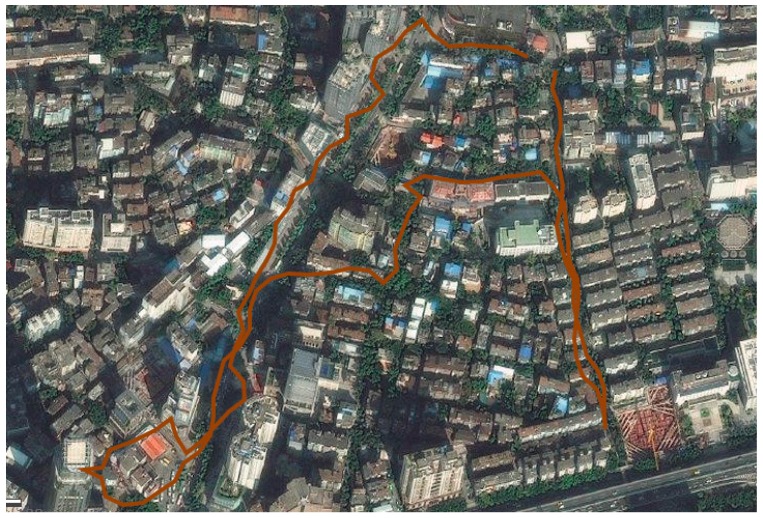
A part of the inspection route of the study site of Sanyu Road, Guangzhou City. The orange lines were inspection routes on an Android mobile device.

Track logs in a GPS Exchange Format (gpx) file contained pictures and videos that were exported to a computer. The track logs (Figure 2) appeared as polylines and were analyzed by Global Mapper 16 [12]. The vertex lists were exported to an Excel workbook to calculate the length and elapsed time of the segment in each premises. The route lengths of inspections in underground parking lots and metros near the entrance were gauged by visual estimation due to a lack of GPS signal in these locations. When an obvious error occurred in a track log between several vertexes, the length was revised by measuring the distance using the measuring tool of Global Mapper.

The infestation levels were estimated by using the container index (CI, percentage of water-holding containers infested with larvae or pupae); the route index (RI, the number of positive habitats per kilometer of inspection route); and the time index (TI, the number of positive habitats per hour of inspection time).

The criteria for vector density control (Mosquito: GB/T 27771-2011) was established by the Chinese National Sanitary City Standard of the National Patriotic Health Campaign Committee (NPHCC), 1 January 2015. Container Index (CI) was abandoned [2] and route index (RI)—the number of positive habitats on a designated route per 1000 m—was used for the justification of reducing mosquito-positive habitats. The RI was divided into grade A (equal to or less than 0.1), grade B (equal to or less than 0.5), and grade C (equal to or less than 0.8). The length of inspection varied with each inspector [10].

Data was analyzed by chi-square (Χ^2^) and Fisher’s exact tests using SPSS 16.0 for Windows (SPSS, Chicago, IL, USA). Premises types were sorted into five broad categories (Table 2): road and around, residential area, underground, farmer’s markets, and other resources. Proportions of all habitat types between the five broad categories of premises types cannot be computed by Fisher’s exact tests with SPSS. Some habitat types and broad categories of premises types were truncated to facilitate statistical analysis.

## 3. Results and Discussion

The total inspection route length and elapsed time for the four study sites was 14.6 km and 10.3 h, respectively (Table 1). Overall, 60 segments were inspected. The total number of potential mosquito habitats was 342, of which 166 were found to have mosquito larvae or pupae, and the total CI was 49% (Table 2). Three mosquito species (*Ae. albopictus*, *Culex quinquefasciatus* Say, and *Culex bitaeniorhynchus* Giles) were collected and identified, of which *Ae. albopictus* was the most common species in small and large containers, stumps, other objects, plastic road barriers, potted or hydroponic plants, fallen leaves, and used tires. Large amounts of *Ae. albopictus* were found in one ditch. Larval *Cx. quinquefasciatus* mosquitoes were found in cisterns, puddles, storm drains, depressions of tree roots, ditches, and subterranean chambers. *Cx. bitaeniorhynchus* Giles was found just once in a ditch with moss. A few *Ae. albopictus* larvae were found with larval *Cx. quinquefasciatus* in underground cisterns, however, *Culex molestus* Forskal was not identified from the samples collected from the underground habitats [13]. Immature mosquitoes were collected from all standing-water habitats in underground parking lots [14]. No positive mosquito habitats were found in the farmer’s market. Only two potential habitats (CI = 50%) were found in the property management residential area. The CI for all habitats and premises ranged from 37% to 67%, except for those with a CI of 0 or 100. There was significant difference in the habitats (Χ^2^ = 27.994, df = 11, *p* < 0.05) and no significant difference in the premises (Χ^2^ = 7.881, df = 10, *p* > 0.05). The CI of underground habitats was significantly (*p* < 0.001) higher than those of the road, the residential area, and other habitats.

Twenty-five mosquito-positive habitats were found in the bird and flower market, which had an RI of 121.5, the highest among all studied premises types, followed by school areas (RI = 73.8), and underground parking lots (RI = 70.9) (Table 2). The RI from the roadside was 4.2, which was 5.3 times higher than the RI of grade C mosquito management criteria created by the NPHCC. The overall RI was 11.3 and the TI was 16.1. The TI in the bird and flower market was the highest (TI = 101.8), followed by the subdivision (TI = 35.3), and school (Table 2).

**Figure 3:**
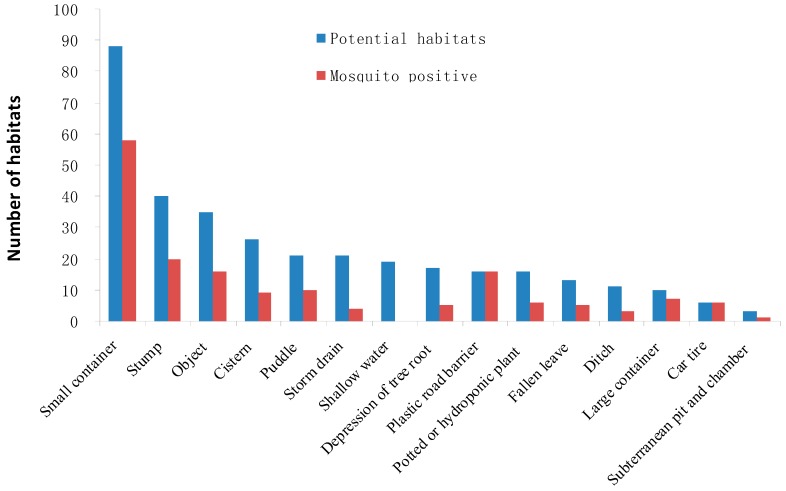
Numbers of potential habitats and mosquito-positive habitats by habitat type in Guangzhou.

Small containers, stumps and other objects were the most abundant potential habitats (Figure 3), accounting for 26% (*n* = 88), 12% (*n* = 40), and 10% (*n* = 35) of the total number, respectively. All 16 plastic road barriers and 6 used tires inspected presented larvae and/or pupae. None of the 19 shallow water habitats were mosquito positive. Immature mosquitoes were found in 66% of small containers, 50% of stumps, 46% of other objects, 35% of cisterns, 48% of puddles, 19% of storm drains, 29% of depressions of tree roots, 38% of potted or hydroponic plants and fallen leaves, 27% of ditches, 70% of large containers, and 33% of subterranean pits and chambers (Χ^2^ = 27.994 df = 11, *p* < 0.01). Immature mosquitoes were significantly more likely to be found in small containers than from other sources like, toys/shoes, cisterns, storm drains, tree roots, potted or hydroponic plants, and ditches (Χ^2^ = 4.261, df = 1, *p* < 0.05).

Urban sources of *Aedes albopictus* are usually refractory to source-reduction in New Jersey, USA [15], however, the source reduction impacted the spatial distribution and abundance of the immature mosquitoes [6,11]. Based on our survey results, the source reduction effort should focus on small containers in Guangzhou City. To our knowledge, the Guangzhou government organized extensive “mosquito fogging” operations in the city to combat the dengue epidemic during mosquito activity seasons, and the larval source reduction responsibilities were given to the owner or manager of a premises. However, the general public lacked motivation, and thought this was the government’s responsibility. The overall RI was 14.2 times more than the grade C criteria of the NPHCC. This suggests that larval source reduction, the key measure of fighting dengue [11,16,17], was not sufficiently carried out in Guangzhou. We suggest that Guangzhou PHCC should promote integrated mosquito management and enhance source reduction for larval control during the adult control operation and urban vector management [2,18]. Since higher RI and/or TI values were in the bird and flower market, school, underground parking lot, and subdivisions, we suggest that the authorities should strengthen the surveillance on these premises types and focus on the mosquito management in wastelands, residential areas, underground structures, bird and flower markets, schools, and driving schools in Guangzhou.

During 2006–2012, Guangzhou Center for Disease Control and Prevention conducted routine *Ae. albopictus* larval surveillance in Guangzhou City using the Breteau index (BI) and CI [19]. The reported number of mosquito-positive habitats in Guangzhou City was underestimated, compared to our study results. This might be caused by inexperienced vector management professionals and different weather conditions. We suggest that the authorities enhance professional training and further study any possible impact factors on immature mosquito population and habitats.

The Android mobile device is a popular and convenient tool. An inspector could quickly gather all field information using these devices after training. Pictures, videos, geographic information of mosquito-positive habitats, and other data could be used for the proof and justification of the mosquito management responsibilities at the premises. Our study provides evidence that immature mosquito habitat surveillance in Guangzhou City, China, using Android mobile devices is feasible and convenient. This method provides much more useful data, compared with pen-and-paper-based field data collection. Lozano-Fuentes et al. (2013) [20] used a cell phone-based system (Chaak) to gather data during the surveillance of immature mosquitoes in the field. The data collected with cell phones was converted to the proper file format and finally it was transferred to a central electronic database. The Android mobile devices with OruxMaps used in our study collected field data more quickly than the cell phone Chaak app, as can be seen by comparing our report with that by Lozano-Fuentes et al. (2013). The application of Android mobile devices (and other new technology developed in the future) will be increased and benefit integrated mosquito management.

## 4. Conclusions

Immature mosquito habitats and the inspection routes were recorded by an Android mobile device with cameras and the OruxMaps and other apps in urban Guangzhou city, southern China, in 2015 after outbreaks of dengue fever in 2014. The total number of potential mosquito habitats was 342, of which 166 (49%) were found with mosquito larvae or pupae. The overall route index (RI) was 11.3, which was 14.2 times higher than the grade C criteria of the National Patriotic Health Campaign Committee (NPHCC). The Android mobile device is a useful tool for surveillance of immature mosquito habitats.

## Figures and Tables

**Table 1:** Length of inspection route and elapsed time in the study sites in Guangzhou, China.

Inspecting Site	Length of Route (km)	Elapsed Time (h)
Sanyu road, Yuexiu District	6.3	3.9
Tangjing street, Baiyun D.	3.3	2.8
Gangwan road, Huangpu D.	2.2	1.6
Fangcun, Liwan D.	2.8	2.1
Total	14.6	10.3

**Table 2:** Number of inspection route segments, number of mosquito-positive habitats, and larval indices in Guangzhou, by premises type.

Premise Type	Number of Segments	Number of Mosquito Positive Habitats	CI (%)	RI	TI
**Road and around**					
Roadside	27	44	42	4.2	8.0
Wasteland	2	6	50	36.5	26.8
Subtotal	29	50	43	4.7	8.7
**Residential area**					
Old residential area	7	17	57	31.4	22.8
Property management residential area	1	1	50	30.3	3.1
Village in city	2	10	50	22.7	35.3
Subtotal	9	28	54	27.6	20.8
**Underground**					
Underground parking lot	3	8	100	70.9	21.3
Metro near the entrance	2	2	100	50.0	30.0
Subtotal	5	10	100	65.4	22.6
**Farmer’s market**	3	0	0	0.0	0.0
**Others**					
Bird and flower market	1	25	52	121.5	101.8
School	1	6	67	73.8	33.9
Driving school	2	7	37	38.8	25.5
Enterprise	2	8	67	25.8	23.8
Park	2	18	49	19.7	19.9
Hospital	3	6	38	18.4	17.9
Hotel	2	8	40	14.9	25.0
Subtotal	13	78	48	30.6	30.1
Total	60	166	49	11.3	16.1

CI = container index, RI = route index, TI = time index.
